# Analysis of Learning Curve in Robot-Assisted Radical Prostatectomy Performed by a Surgeon

**DOI:** 10.1155/2020/9191830

**Published:** 2020-05-26

**Authors:** Roman I. Slusarenco, Konstantin V. Mikheev, Artem O. Prostomolotov, Roman B. Sukhanov, Evgeny A. Bezrukov

**Affiliations:** ^1^Institute for Urology and Reproductive Health, Moscow, Russia; ^2^International School “Medicine of the Future”, FGAOU VO I.M. Sechenov First Moscow State Medical University, Moscow, Russia

## Abstract

This study aimed to report the learning curve in robot-assisted radical prostatectomy (RARP) performed by one surgeon who is experienced in laparoscopic prostatectomies. The records of 145 RARP cases performed between 2015 and 2017 were evaluated retrospectively. Patients were divided into three groups: group 1 comprised the first 49 cases, group 2 comprised 50–88 cases, and the rest of the cases were assigned to group 3. Continence was defined as the necessity to use at least one pad during a day. Additionally, erectile function recovery was defined as having erection sufficient for sexual intercourse regardless of using a phosphodiesterase type 5 inhibitor. Continence and erectile function recovery were assessed during interviews at 3, 6, and 12 months after surgery. First, all procedures were successfully performed without conversions or blood transfusions. The median follow-up period was 22 months. Moreover, the median skin-to-skin operative time (OT) was 220 minutes. The median blood loss was 150 ml, and the mean hospital stay was 8.9 ± 3.87 days. The median prostate volume was 36 cm³. The overall positive surgical margin rate was 13.1%. Overall, 38 (26.2%) postoperative complications were observed, and 17.9% of them were graded as minor. Anastomotic leakage decreased significantly from group 1 to group 3 (26.5% and 7%, respectively). The continence recovery (0-1 pad) rates were 60.6%, 75.7%, and 84.9% at 3, 6, and 12 months after surgery, respectively. Subsequently, the erectile function recovery rates were 50.9% and 65.4% at 6 and 12 months after surgery, respectively. In conclusion, there are several types of learning curves for RARP. First, the shallowest learning curve was observed for the OT. Regarding the analysis of “advanced learning curve,” demonstrating the improvement of OT and blood loss is considered insufficient. Therefore, additional oncological and functional results that require a longer period of investigation are required.

## 1. Introduction

Prostate cancer is the second most common type of cancer and is the fifth leading cause of oncological death in men worldwide [1]. Prostatectomy is the gold standard treatment option for patients with localized disease [2]. Previously, this procedure was performed with a retropubic open approach [3]. The main drawbacks of this approach are as follows: long hospitalization, necessity of prescribing additional pain medication, and significant blood loss. Today, mini-invasive methods, such as laparoscopic radical prostatectomy (LRP) and robot-assisted radical prostatectomy (RARP), are becoming significantly more popular compared with the retropubic open approach. In particular, RARP has replaced retropubic radical prostatectomy (RRP) in Europe and the USA, which has been the gold standard surgical treatment of the localized and locally advanced prostate cancer [2]. Clearly, RARP has become the main standard treatment of prostate cancer. Recently, RARP has also demonstrated better outcomes than RRP and LRP. Although RARP has several advantages, adequate equipment and sufficient training programs are still required in this treatment method. Thus, this technique is rarely used in developing countries considering that it lacks financial support [4].

A short learning curve is one of the main advantages of the RARP, which makes it an interesting option for junior doctors. Certainly, surgeons who do not have significant experience in laparoscopic surgery achieve promising results. According to a recent study, doctors perform RARP more quickly compared with laparoscopic techniques [5]. Additionally, decreased intraoperative blood loss and reduced number of complications are observed after performing a total of 25–40 RARPs [6, 7]. Nevertheless, a significant clinical experience is still required to achieve adequate functional and oncological results in terms of negative surgical margins when performing RARP [8]. There are two types of learning curves, which can be used for RARPʼs assessment [9, 10]. The “basic learning curve” includes 25–50 cases, after which the operative time (OT) decreases to 200–240 minutes [9]. In case of the “advanced learning curve,” 100–300 cases are required to achieve stable erectile function and continence recovery [9]. There are several factors that affect the speed of surgical technique mastery including the following: initial surgical skills, type of the learning program, and experience in performing laparoscopic surgeries [11, 12]. This study aimed to identify the pace of mastering RARP by a surgeon with significant clinical experience in performing LRP using the structured “advanced learning curve” method.

## 2. Materials and Methods

We studied 145 patients who had undergone radical robotic prostatectomy. It is worth mentioning that only one surgeon who is experienced in laparoscopic surgery had been performing RARP between 2015 and 2017. The data were collected retrospectively. All patients were divided into three groups according to the surgery date: group 1 comprised the first 49 cases, group 2 comprised 50–88 cases, and the rest of the cases were assigned to group 3.

All patients underwent standard transperitoneal RARP. Pelvic lymph node dissection (PLND) was performed in case of possible lymph node involvement according to the Briganti nomogram [13]. Moreover, a nerve-sparing technique was performed when prostate cancer was localized according to the D'Amico classification. The postoperative general prostate-specific antigen (PSA) level was measured at 3, 6, 9, and 12 months after surgery. According to the recommendations, biochemical recurrence was considered provided that the general PSA level was higher than 0.2 ng/ml [14]. Positive surgical margin (PSM) was defined as a tumor observed at the inked margin. Patients experiencing the extension of the tumor through the prostatic capsule were considered to experience extracapsular extension (pT3).

We considered our patients continent if they used a maximum of 1 pad per day. Continence was evaluated at 3, 6, and 12 months after surgery. Erectile function was evaluated at 6 and 12 months after surgery using the International Index of Erectile Function-5 (IIEF-5) score. Erectile function recovery was defined as the ability to achieve a sufficient erection for sexual intercourse regardless of using a phosphodiesterase type 5 (PDE 5) inhibitor. The initial results included OT (in minutes), positive surgical margins, blood loss volume, duration of urinary bladder catheterization, and continence and sexual function's recovery. Statistical analysis was performed using the International Business Machines Corporation Statistical for the Social Sciences Statistics version 23. *p* < 0.05 was considered statistically significant.

## 3. Results

There were no statistically significant differences between the three groups in terms of age, body mass index, IIEF-5 total score, PSA level, prostate volume, Gleason score, clinical stage, DʼAmico oncological risk, and possibility of lymph node invasion according to the Briganti nomogram (Table 1).

**Figure 1:**
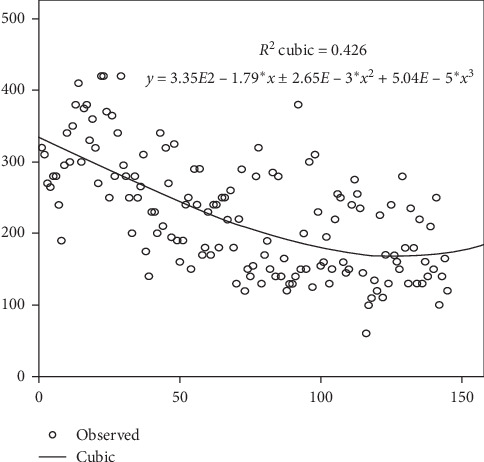
Learning curve for the operative time.

The median follow-up periods for groups 1, 2, and 3 were 24, 22, and 20 months, respectively (*p* < 0.001). The median age of the patients was 62.18 ± 6.76 years. The mean PSA level was 9.24 ± 6.86 ng/ml. The median OT (defined as the time from creating the first incision to creating the final skin suture, including the time of docking and undocking) was 220 (150–280) minutes. The OT decreased significantly from group 1 to group 3 (*p*=0.0001) (Figure 1).

**Figure 2:**
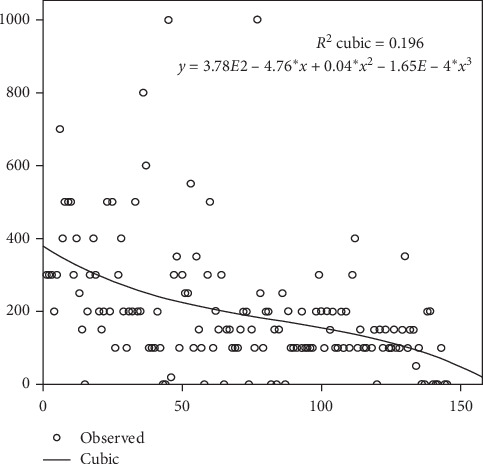
Learning curve for the blood loss.

Simultaneously, none of the patients required conversion. The median blood loss was 150 (100–250) ml. As the surgeon gained experience, blood loss decreased: group 1, 250 (150–400) ml; group 2, 150 (100–250) ml; and group 3, 100 (100–175) ml (*p*=0.0002). None of the patients required blood transfusion (Figure 2).

Overall, 38 (26.2%) postoperative complications were observed using the Clavien–Dindo classification, and 26 of them (17.9%) were graded as minor (Clavien I/II) (Table 2).

Clavien IV/V complications were not observed. The incidence rates of anastomotic leakage that required prolonged catheter duration were 26.5%, 23.1%, and 7.0% in groups 1, 2, and 3, respectively (*p* < 0.02). Six major urine extravasations, which required unilateral or bilateral ureteral mono-J stent placement, were observed in the early postoperative period. Six patients required cystoscopy and repeat catheter placement due to major urine extravasation after catheter removal (Table 3).

Extended PLND was performed in only 38 patients (26%). A total of nine patients from group 3 underwent pelvic lymphadenectomy up to the level of the abdominal aorta bifurcation (Table 4).

According to the histological investigation results, the Gleason total score and pT stage were equal in all the three groups (*p* > 0.05). Extracapsular invasion (pT3) and seminal vesicle invasion (pT3b) were observed in 13.79% and 7.6% of patients, respectively. The overall PSM rate was 13.1%, corresponding to 8.2% in pT2 and 19.7% in pT3. A comparison of the pT2 (*p*=0.065), pT3 (*p*=0.162), and the overall groups (*p*=0.07) showed no statistically significant difference (Table 5).

We considered our patients continent if they used a maximum of 1 pad per day. Continence was defined as the necessity to use one pad per day. Thus, at 3, 6, and 12 months after surgery, the overall continence rates were 60.6%, 75.7%, and 84.9%, respectively. In comparison with urinary incontinence among the observed groups, at 3 months after surgery, the rates were 64.5%, 66.7%, and 53.5%, respectively (*p*=0.146), and there was no statistically significant difference between the three groups. At 6 months after surgery, continence was completely restored in 77.4%, 86.7%, and 66.7% of patients, respectively (*p*=0.034). At 12 months after surgery, 83.0%, 89.6%, and 82.1% of patients were continent in groups 1, 2, and 3, respectively (*p*=0.237), with no statistically significant difference between the three groups. Erectile function was considered to be restored if erection was sufficient for sexual intercourse regardless of using a (PDE 5) inhibitor. Hence, erectile function recovery was achieved in 50.9% (IIEF-5 score, 13.9) and 65.4% (IIEF-5 score 15.3) of patients at 6 and 12 months after surgery, respectively (*p*=0.176). There were no statistically significant differences in the observed groups.

## 4. Discussion

RARP has become the most common surgical procedure that is used in the treatment of localized prostate cancer. Surgeons prefer RARP over other surgical methods because of its mini-invasive characteristics and short learning curve. Patel et al. found that only 25 surgeries have to be performed to master RARP [7].

Nevertheless, each surgeon has his/her own learning curve and needs to perform an individual number of surgeries to achieve desirable results.

The main intraoperative index is OT, which is changing as long as the surgeon masters the RARP technique. Long operations are possibly associated with technical difficulties and lack of surgical skills [15]. In our study, the median (skin to skin) OT (interquartile range (IQR)) was 220 (150–280) minutes, which decreased after the 88-th case. According to Doumerc et al., if a surgeon had experience in performing open prostatectomies, 110 cases would be required to achieve the OT of 180 minutes [16]. Our results are consistent with the results of Haglind et al.'s study, which demonstrated that the median OT in the group of RARP was 236 minutes.

The median blood loss was 150 (100–250) ml. Evidently, it decreased after completing a hundred of cases by 100 ml in group 3 (median level, IQR). According to the statistical data, blood loss rates during RARP vary from 142 ml to 230 ml [17], which is considered another advantage of RARP over open radical prostatectomy. According to Tobias-Machado et al., the median blood loss during the first 60 robot-assisted radical prostatectomies was 245.5 ml, as the surgeon had already performed 200 laparoscopic radical prostatectomies [18]. In our investigation, the median hospital stay was 8.9 ± 3.87 days although Rocco et al. mentioned the 3-day hospital stay [19]. Certainly, hospital stay is longer in Russia than that in the recent study considering the differences between the two healthcare systems.

The overall complication rate was 26.2%. Particularly, the complication rates were 17.9% and 8.3% for Clavien I/II and Clavien IIIA complications, respectively. According to Rocco et al., the overall anastomotic leakage rate was 24%, with the anastomoses diagnosed by the postoperative retrograde cystogram. These results could be compared with our data (median rate of 17.9%) [19]. Moreover, the anastomotic leakage rate was evidently lower as the surgeon was gaining experience than that of group 1 (26.5% and 7%, respectively).

Finally, according to Hruza et al., the complication rates were 21.7% for Clavien I/II and 11.5% for Clavien III [20].

Certainly, the main goal of surgical treatment is to achieve the optimal oncological results. Hence, the PSM was the most important rate. According to the systematic review by Yossepowitch et al., the overall PSM rate in different studies varies from 6.5% to 32% [21]. In our investigation, the overall PSM rate was 13.1%, and the PSM rates in pT2 and pT3 were 8.2% and 19.7%, respectively. These results are consistent with the abovementioned results. Interestingly, the PSM rate did not change alongside the learning curve evidently, suggesting that a surgeon should continue to master one's skills after performing 150 surgeries. Some investigators believe that the PSM rate did not change alongside the learning curve because the tipping point might have not been achieved yet [22].

We also believe that the data obtained from Patel et al. should be paid careful attention because they showed that PSM could significantly change after performing 1500 surgeries (e.g., 12.2% for 1–300 cases, 6.6% for 301–600 cases, 13.6% for 601–900 cases, 11% for 901–1.200 cases, and 1.8% for 1201–1500 cases) [23].

Biochemical recurrence was observed in 16.5% of patients at the end of the study period.

Ploussard et al. [24] determined that the overall continence rates (unnecessary use of pads after RARP) were 50.3%, 72%, and 83.6% at 3, 6, and 12 months after surgery, respectively. This result is consistent with the result of our present study (continence rates of 60.6%, 75.7%, and 84.9% at 3, 6, and 12 months after surgery, respectively).

Another important life quality rate is erectile function. Erectile function recovery rates at 6 and 12 months after surgery were 50.9% (IIEF-5 score, 13.9) and 65.4% (IIEF-5 score, 15.3), respectively. In one study (Ploussard et al.), 42% and 57.7% of patients demonstrated adequate erectile function at 6 and 12 months after surgery, respectively [24]. Kim et al. reported that erectile function was restored in 33% and 57.1% of patients at 3 and 6 months, respectively, in the large RARP group (*n* = 528) [25].

Our investigation has a number of limitations. First, this was a nonrandomized and retrospective study. Second, a short period of supervision was observed in this study. The results were assessed throughout the 12-month period after surgery. In RARP, the skills of a surgeon should be developed constantly. Third, small sample sizes were observed in the three groups, decreasing the sample value. Finally, continence and erectile function recovery rates were evaluated through patient interviews without the use of questionnaires.

During the whole learning curve, we observed the considerable improvement of the following rates: OT, blood loss volume, hospital stay duration, anastomotic leak frequency after surgery (according to the cystographic results), and frequency of abdominal cavity draining. Simultaneously, a longer period of investigation and manual practicing are required to evaluate PSM and BCR and urine continence and sexual function recovery.

## 5. Conclusions

In conclusion, the results of our study have provided evidence that surgeons with previous surgical experience require more than 80 cases to achieve an OT of 180 minutes. The median blood loss, which was approximately 150 ml, was achieved approximately after performing 50 surgeries. Moreover, the PSM had not significantly changed after performing 145 surgeries. Regarding urinary incontinence and erectile function, surgeons started performing simple surgeries and eventually managed to perform nerve-sparing techniques with anterior and posterior reconstructions for better clinical outcomes.

## Figures and Tables

**Table 1:** Preoperative clinical characteristics of the patients.

Characteristics	Median value	Group 1 (1–49)	Group 2 (50–88)	Group 3 (89–145)	*p* value
Age (years)					
Mean ± SD	62.18 ± 6.76	61.29 ± 6.73	63.56 ± 6.75	62.00 ± 6.76	0.42
Median (IQR)	62 (57.5–67)	62 (57–67)	63 (60–68)	62 (57–66.5)	
BMI					
Mean ± SD	27.24 ± 3.85	26.76 ± 3.91	27.28 ± 4.71	27.64 ± 3.11	0.52
Median (IQR)	27.0 (25.0–28.7)	27.1 (24.6–28.5)	26.5 (24.6–29.0)	27.0 (25.6–29.0)	
IIEF-5, *n* (%)					
21–25 points	60 (53.1)	17 (53.1)	13 (46.4)	30 (56.6)	0.35
16–20 points	24 (21.2)	8 (25.0)	4 (14.3)	12 (22.6)	
11–15 points	10 (8.8)	4 (12.5)	2 (7.1)	4 (7.5)	
0–10 points	19 (16.8)	3 (9.4)	9 (32.1)	7 (13.2)	
PSA (ng/ml)					
Mean ± SD	9.24 ± 6.86	8.48 ± 5.97	8.71 ± 5.65	10.25 ± 8.19	0.553
Median (IQR)	7.47 (5.5–9.8)	7.47 (5.6–9.1)	6.97 (5.3–9.9)	8.00 (5.7–10.6)	
Prostate volume (cm^3^)					
Mean ± SD	39.84 ± 17.14	38.53 ± 17.57	41.74 ± 20.87	39.58 ± 13.82	0.55
Median (IQR)	36.0 (29.0–47.5)	33.0 (26.5–49.5)	34.00 (29–50)	40 (30.00–45.5)	
Clinical stage, *n* (%)					
сТ1	90 (62.1)	34 (69.4)	23 (59.0)	33 (57.9)	0.8
сТ2	40 (27.6)	11 (22.4)	13 (33.3)	16 (28.1)	
сТ3	15 (10.3)	4 (8.2)	3 (7.7)	8 (14.0)	
Biopsy Gleason score, *n* (%)					
≤6	89 (61.4)	32 (65.3)	28 (71.8)	29 (50.9)	0.08
=7	48 (33.1)	17 (34.7)	10 (25.6)	21 (36.8)	
≥8	8 (5.5)	0 (0)	1 (2.6)	7 (12.3)	
D'Amico risk, *n* (%)					
Low	78 (53.8)	28 (57.1)	29 (74.4)	21 (36.8)	0.128
Median	38 (26.2)	14 (28.6)	5 (12.8)	19 (33.3)	
High	29 (20)	7 (14.3)	5 (12.8)	17 (29.8)	
Briganti lymph node invasion, *n* (%)					
<5%	92 (63.4)	34 (69.4)	26 (66.7)	32 (56.1)	0.33
≥5%	53 (36.6)	15 (30.6)	13 (33.3)	25 (43.9)	

**Table 2:** Intraoperative data.

Characteristic	Median (total)	Group 1 (1–49)	Group 2 (50–88)	Group 3 (89–145)	*p*
Operative time (OT) (min)					
Mean ± SD	224.17 ± 80.87	293.57 ± 68.81	204.23 ± 57.48	178.16 ± 62.22	0.0001
Median (IQR)	220 (150–280)	295 (250–340)	190 (150–250)	160 (130–227.50)	
Console time (min)					
Mean ± SD	120.45 ± 56.40	163.06 ± 58.26	107.44 ± 38.16	92.72 ± 42.35	0.0002
Median (IQR)	100 (80–155)	160 (115–210)	100 (80–130)	80 (70–110)	
Blood loss (mL)					
Mean ± SD	199.10 ± 175.09	290.20 ± 206.8	189.74 ± 182.1	127.19 ± 84.02	0.0002
Median (IQR)	150 (100–250)	250 (150–400)	150 (100–250)	100 (100–175)	
Hospital stay (days)					
Median (IQR)	11 (8.5–14)	12 (9–16)	11 (7–15)	10 (8–12.5)	0.007
Abdominal drains, *n* (%)					
Placed	69 (47.6)	39 (79.6)^*∗*^	21 (53.8)	9 (15.8)^*∗*^	0.0001
Not placed	76 (52.4)	10 (20.4)	18 (46.2)	48 (84.2)	
Complications, *n* (%)					
Clavien grade I/II	26 (17.9)	13 (26.5)^*∗*^	9 (23.1)	4 (7.0)^*∗*^	0.02
Clavien grade III	12 (8.3)	8 (16.3)^*∗*^	2 (5.1)	2 (3.5)^*∗*^	0.116

^*∗*^Statistically significant difference.

**Table 3:** Complications stratified by the Clavien–Dindo classification system.

Grade	Complications	Group 1 (1–49)	Group 2 (50–88)	Group 3 (89–145)	Management
I/II	Anastomosis leakage	13	9	4	Prolonged catheter duration

IIIA	Major urine extravasation during catheterization	3	1	2	Ureteral stent (mono-J stent)

IIIA	Major urine extravasation after catheter removal	5	1	0	Cystoscopy and catheter placement

**Table 4:** Operative technique.

Characteristic	Total	Group 1 (1–49)	Group 2 (50–88)	Group 3 (89–145)	*p*
Nerve sparing, *n* (%)					
Nonnerve sparing	56 (38.6)	14 (8.6)	17 (43.6)	25 (43.9)	0.035
Bilateral	73 (50.3)	27 (55.1)	15 (38.5)	31 (54.4)	
Unilateral	14 (9.7)	7 (14.3)^*∗*^	7 (17.9)	0 (0.0)^*∗*^	
Partial bilateral	2 (1.4)	1 (2.0)	0 (0.0)	1 (1.8)	
Prostatectomy technique, *n* (%)					
Intrafascial	72 (49.7)	25 (51)	15 (38.5)	32 (56.1)	0.023
Interfascial	8 (5.5)	6 (12.2)^*∗*^	2 (5.1)	0 (0)^*∗*^	
Extrafascial	59 (40.7)	15 (30.6)	19 (48.7)	25 (43.9)	
Combined technique	6 (4.1)	3 (6.1)	3 (7.7)^*∗*^	0 (0)^*∗*^	
Prostate venous plexus (PVP) suture ligation, *n* (%)					
No suture ligation	21 (14.5)	3 (6.1)^*∗*^	10 (25.6)^*∗*^	8 (14)	0.034
Suture ligation before transection	83 (57.2)	32 (65.3)^*∗*^	15 (38.5)^*∗*^	36 (63.2)	
Suture ligation after transection	41 (28.3)	14 (28.6)	14 (35.9)	13 (22.8)	
Anterior reconstruction					
Not performed	66 (45.5)	20 (40.8)	24 (61.5)^*∗*^	22 (38.6)^*∗*^	0.045
Performed	79 (54.5)	29 (59.2)	15 (38.5)^*∗*^	35 (61.4)^*∗*^	
Posterior reconstruction, *n* (%)					
Performed	26 (17.9)	12 (24.5)	6 (15.4)	8 (14.0)	0.33
Not performed	119 (82.1)	37 (75.5)	33 (84.6)	49 (86.0)	
Pelvic lymph node dissection (PLND), *n* (%)					
Not performed	107 (73.8)	36 (73.5)	32 (82.1)	39 (68.4)	0.003
Up to the ureter level	29 (20.0)	13 (26.5)	7 (17.9)	9 (15.8)	
Up to the aorta bifurcation level	9 (6.2)	0 (0)^*∗*^	0 (0)	9 (15.8)^*∗*^	

^*∗*^Statistically significant difference.

**Table 5:** Histological results.

Characteristics	Total	Group 1 (1–49)	Group 2 (50–88)	Group 3 (89–145)	*p*
Pathological stage (pT), *n* (%)					
pT2	125 (86.21)	41 (83.67)	34 (87.18)	50 (87.72)	0.51
Postoperative Gleason score, *n* (%)					
≤6	65 (44.8)	25 (51.0)	18 (46.2)	22 (38.6)	0.2
7	71 (49.0)	24 (49.0)	17 (43.6)	30 (52.6)	
≥8	9 (6.2)	0 (0.0)	4 (10.3)	5 (8.8)	
Invasion, *n* (%)					
No invasion (pT2)	102 (70.3)	36 (73.5)	32 (82.1)	34 (59.6)	
Capsular infiltration (pT2)	23 (15.9)	5 (10.2)	3 (7.7)	16 (28.1)	0.097
Extracapsular invasion (pT3)	20 (13.79)	8 (16.33)	5 (12.82)	7 (12.28)	
Seminal vesicle (pT3b)	11 (7.6)	3 (6.1)	3 (7.7)	5 (8.8)	
Surgical margin status, *n* (%)					
Positive	19 (13.10)	3 (6.12)	9 (23.08)	6 (10.53)	0.07
Negative	126 (86.9)	46 (93.88)	30 (76.92)	51 (89.47)	

## Data Availability

The data used to support the findings of this study are available from the corresponding author upon request.
